# Localized corrosion in selective laser melted SS316L in CO_2_ and H_2_S brines at elevated temperatures

**DOI:** 10.1038/s41529-024-00468-4

**Published:** 2024-05-10

**Authors:** Deeparekha Narayanan, Alan Martinez, Ulises Martin, Bilal Mansoor, Raymundo Case, Homero Castaneda

**Affiliations:** 1 Department of Materials Science and Engineering, Texas A&M University, College Station, TX 77843 USA; 2 Texas A&M University at Qatar, Doha, Qatar

**Keywords:** Metals and alloys, Corrosion

## Abstract

In this work, the passivation and localized corrosion of selective laser melted (SLM) stainless steel 316 L when exposed to high pressures of CO_2_ with the presence of H_2_S and Cl^−^ at 25 °C and 125 °C were studied. Depletion of Cr/Mo was observed at the cell interiors and melt-pool boundaries (MPBs) compared to the cell boundaries. Volta potential differences obtained from scanning Kelvin probe force microscopy (SKPFM) showed that the MPBs were 8–20 mV lower than the matrix, while the cell interiors were 20–50 mV lower than the cell boundaries. Electrochemical impedance spectroscopy (EIS) and Mott–Schottky tests indicated a more defective passive film at 125 °C, and X-ray photoelectron spectroscopy (XPS) confirmed the formation of a less protective film with an increased S/O ratio at 125 °C than 25 °C. Initiation of localized corrosion was observed at the MPBs and pits formed after a week of immersion were wider by an order of magnitude at 125 °C than 25 °C, with evidence of cell-interior dissolution. While passivity was observed even at elevated temperatures, local chemical heterogeneities compromised the stability of the film and contributed to localized corrosion in SLM SS316L.

## Introduction

Austenitic stainless steel 316L (SS316L) is widely used in the oil and gas industry due to its excellent corrosion resistance and desirable mechanical properties. Due to its innate ability to passivate, it has been proven to be reliable as piping and cladding material for condensed natural gas environments with CO_2_ and S containing species including H_2_S, S_2_O_3_^2−^, SO_3_^2−^, and HS^−^^1,2^. Selective laser melting (SLM) is a popular additive manufacturing (AM) technique used to manufacture intricate three-dimensional parts with low defect densities, finer microstructures and improved corrosion resistance^3^. Comparisons of the corrosion performance of wrought and AM stainless steels have been studied extensively in different types of media, including chloride-containing solutions^4–8^, biomedical^9,10^, and acidic environments^11,12^ with the enhanced corrosion resistance of the SLM alloys being well documented in ambient testing conditions. The finer structures formed (about two orders of magnitude lower than wrought microstructures), absence of anodic MnS inclusions and the dislocation networks have been stated as the reason for the formation of a dense, protective passive film with lower defect densities^4,13^. The formation of dislocation networks at the cell boundaries and the enrichment of Cr/Mo at these regions have been reported by several works^5,12–17^, leading to a competing effect on whether the cell interiors or boundaries become the preferred site of localized attack. There is however a discrepancy regarding which microstructural features were more susceptible to attack, with different studies reporting these to be the melt-pool boundaries (MPBs)^8,16,18^, cell boundaries^12,19^, inclusions^12,20^ as well as cell interiors^15,17^. Most of the studies performed showed corroded surfaces or pits formed when higher potentials were applied to accelerate the film breakdown and localized corrosion. Depending on the potential limits set in the cyclic polarization tests, test scan rates, and printing parameters, the results obtained will vary accordingly.

Reference ^11^ proposed that the SLM SS316L which had its native oxide film intact, showed dissolution of the inclusions rich in Mn, Si, and O at potentials close to the open circuit potentials with subsequent cell boundary attack upon polarization to transpassive potentials. However, for the samples that were cathodically polarized to remove native oxide film before the tests, lower potentials led to cell-interior dissolution and transpassive potentials caused cell boundary attack. Therefore, they proposed a competing effect between the high Cr/Mo at the boundaries which rendered them more cathodic at lower potentials, versus the increased dissolution of Cr to Cr^6+^ at the boundaries, making them more susceptible to attack. While SLM SS316L components with purely dislocation structures at the cell boundaries and no Cr/Mo segregation have been reported^21^, it is evident that the Cr/Mo segregation is the reason for increased protection of these features as the high dislocation density would ideally cause increased corrosion of these regions^22^. However, in tests conducted by ref. ^23^ on SLM SS316L in molten salt conditions at 650 and 700 °C, they showed homogenous corrosion independent of the aforementioned features. While these nano-scale segregations did not contribute to any localized attack, the presence of more cell boundaries and dislocation networks in the SLM microstructure aided in increased Cr diffusion to the surface, hence forming a protective Cr_2_O_3_ film that prevented the intergranular attack exhibited by the wrought counterpart. Therefore, the corrosion behavior and the effect of the features developed in SLM SS316L is highly dependent on the nature of testing and how they interacted with the environment. Since the body of literature studying these materials at extreme conditions is limited, especially in simulated deep well environments that contain high pressures of CO_2_, H_2_S, Cl^−^ and high temperatures, it becomes important to understand if SLM SS316L exhibits the same enhanced corrosion behavior in these environments as well. Additionally, since the manufactured materials are typically not polarized when in service, it is also necessary to study whether these features are involved in the corrosion process under immersion conditions as well.

In H_2_S environments, temperature has been shown to have detrimental effects in the passive film performance^24,25^. The presence of corrosion products on stainless steel samples upon exposure to H_2_S and Cl^−^ containing environments indicates the breakdown of passivity^25–27^. The passive films formed on stainless steels in H_2_S-containing environments are more defective, with lower charge transfer resistances, when compared to H_2_S-free electrolytes^1^. Reference ^25^ proposed that increased S adsorption and the competing effect between S and O to occupy the anion vacancies in the barrier layer at higher temperatures caused a reduction in protective oxides and promoted sulfide formation. Additionally, the depletion of Cr in the passive film as well as a reduction in the Cr_2_O_3_ content was apparent in stainless steel in CO_2_ saturated environments, leading to increased dissolution kinetics^28,29^. Though the effects of CO_2_ and H_2_S have widely been described in wrought and cast steels, there is a lesser understanding of the passivation capabilities, passive film composition and pit initiation mechanisms of SLM SS316L in these types of environments.

In this study, effect of elevated temperature on the passivation and localized corrosion behavior of SLM SS316L was studied in a simulated sour environment containing 3500 kPa CO_2_, 4.6×10^−3^ mol% H_2_S in the aqueous phase (=0.1 mol% H_2_S in gas phase at 25 °C) and 3.5 wt% NaCl. The test temperatures selected were 25 and 125 °C with a lower temperature selected as a control to compare the behavior at elevated temperature against. The microstructure of the SLM SS316L was characterized using techniques such as optical microscopy (OM), scanning electron microscopy (SEM) and scanning transmission electron microscopy (STEM). The electrochemical characteristics of the microstructure were characterized using scanning Kelvin probe force microscopy (SKPFM) to study any Volta potential differences between the features developed. To study the corrosion resistance, the ability of the alloys to passivate and the electrochemical characteristics of the film formed, electrochemical impedance spectroscopy (EIS) and Mott–Schottky tests were performed. The composition of the film formed was characterized by X-ray photoelectron spectroscopy (XPS) after one day of immersion. After one day as well as one week of exposure, the surfaces were characterized using SEM to study instances of localized corrosion. A correlation between the various microstructural features and the mechanism of localized corrosion occurring upon immersion was developed. This work aims to add to the limited knowledge of the corrosion behavior of SLM SS316L in simulated deep well environments.

## Results and discussions

### Initial microstructural characterization

**Fig. 1:**
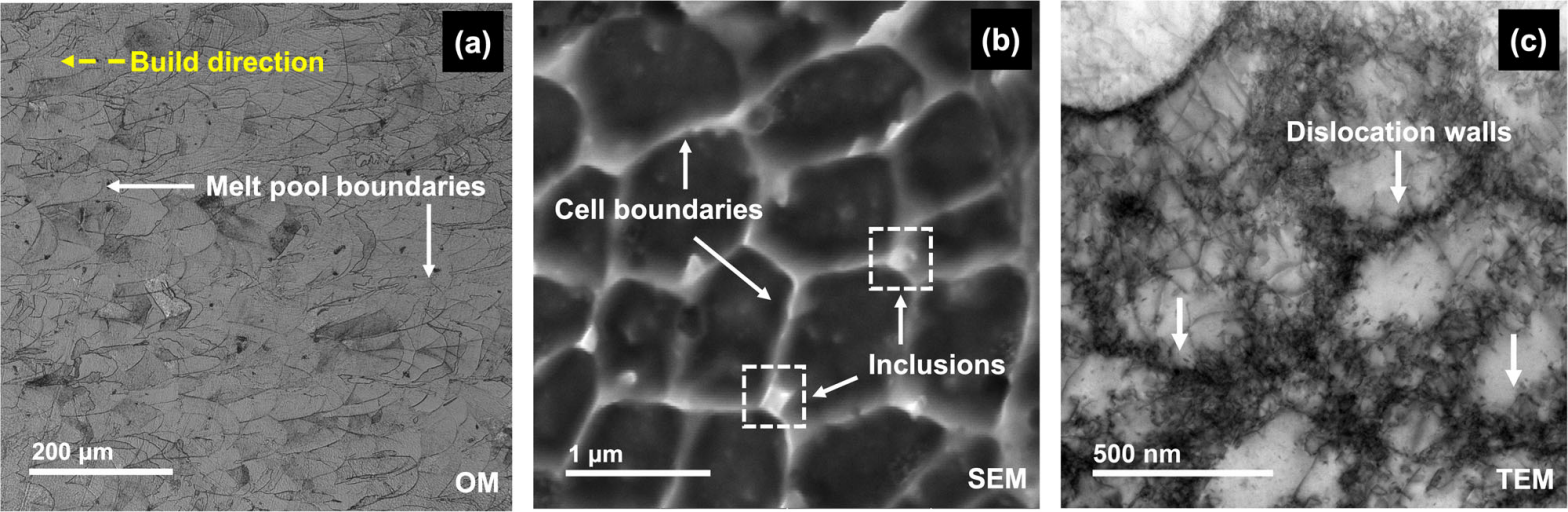
Microstructural features present in the SLM SS316L sample. **a** Optical microscopy (OM) image of the test surface along the build direction showing the melt-pool boundaries (MPBs) formed (magnification = 100×); **b** Scanning electron microscope (SEM) image showing the cellular structures, cell boundaries and nanoinclusions (Magnification = 58, 800×); **c** TEM image showing the formation of dislocation walls along the cell boundaries.

**Fig. 2:**
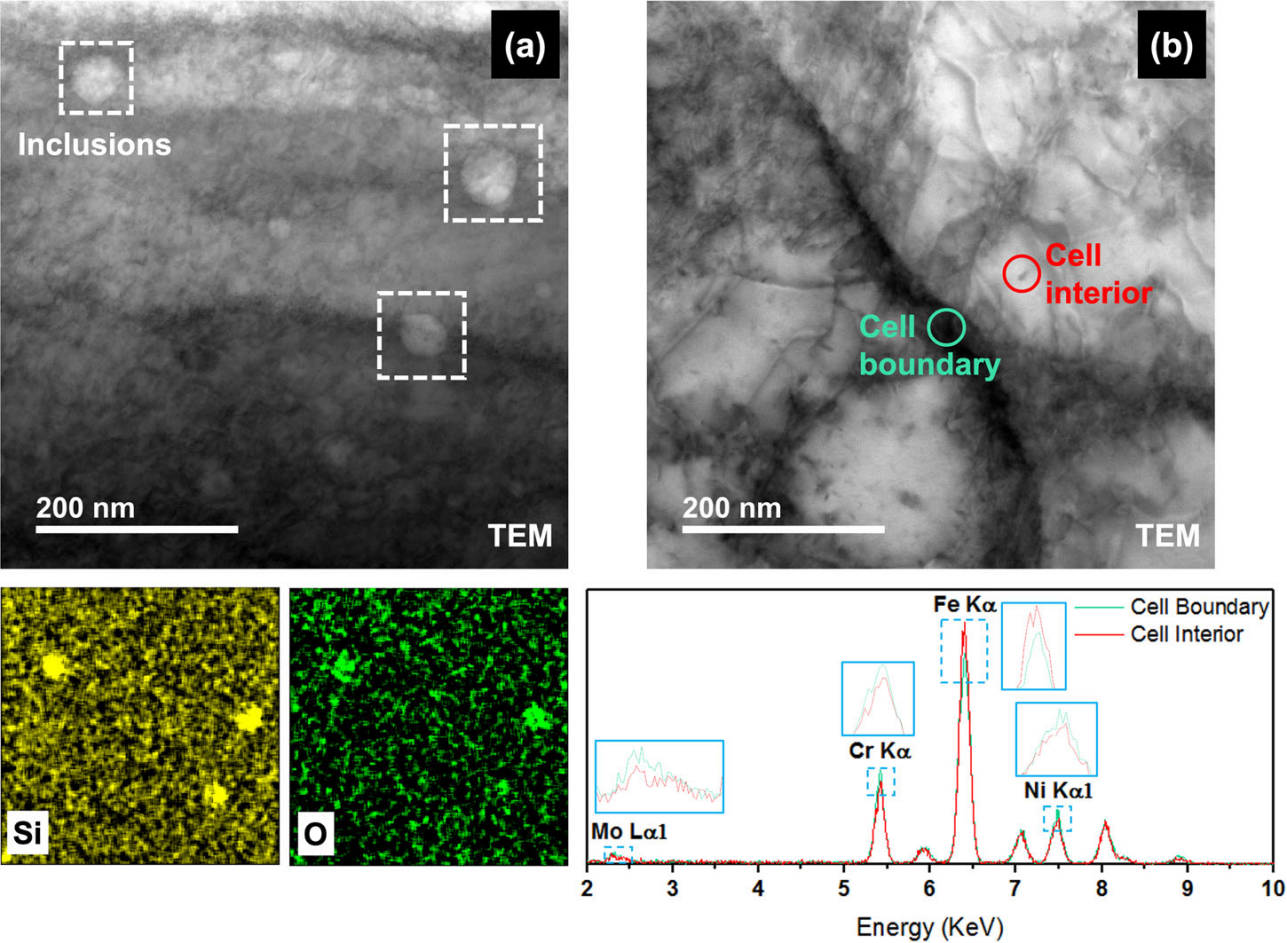
Elemental analysis of microstructural features. **a** TEM-EDS maps showing the silicon oxide nanoinclusions and **b** TEM-EDS spectra at the cell interior and boundary showing increased Cr, Ni, and Mo at the cell boundaries and reduced Fe in comparison to the cell interiors.

Figure 1(a) shows the optical microscopy (OM) image of the test surface. Semi-elliptical melt-pool boundaries (MPBs) were formed due to the melting and fusing of the powder bed by the scanning laser. Since the surface tested was along the build direction, the presence of higher density of MPBs is expected when compared to the surface perpendicular to the build direction. Energy dispersive spectroscopy (EDS) line scans of the areas with the MPBs showed a slight drop of Cr and Mo at these regions when compared to the matrix (shown in Supplementary Fig. 1). A mixture of various cell types such as columnar, equiaxed and columnar dendritic was found to be present across the surface depending on the mode of heat transfer. Figure 1(b) shows the cellular structures formed (of width 0.5–2 μm) in the SLM SS316L under the SEM with cell boundaries containing some nanoinclusions. The presence of dense dislocation networks at the cell boundaries can be observed through higher magnification imaging under the transmission electron microscope (TEM) (Fig. 1c) which has been attributed to the high localized stresses caused during the rapid solidification occurring during the SLM process^30^. These nanoinclusions were identified to be silicon oxide and not anodic MnS inclusions through TEM-EDS maps (Fig. 2a) that are present in wrought SS316L which have also been reported by ref. ^3^. These inclusions are formed because of the presence of Si which has a high tendency to oxidize and is added to the feedstock powder to improve the flowability of the melt^31^. Through TEM-EDS point scans at the cell interior as well as boundaries (Fig. 2b), it is evident that there is a higher content of Cr, Ni, and Mo at the cell boundaries with a depletion of Fe compared to the cell interiors. The distribution obtained was found to be similar to that reported by ref. ^12^ for SLM SS316L. This solute segregation has been reported to be a characteristic of most SLM-manufactured components due to the rejection of the solute to the solid/liquid interface due to the lower solubility in the solvent in the solid phase^32^.

**Fig. 3:**
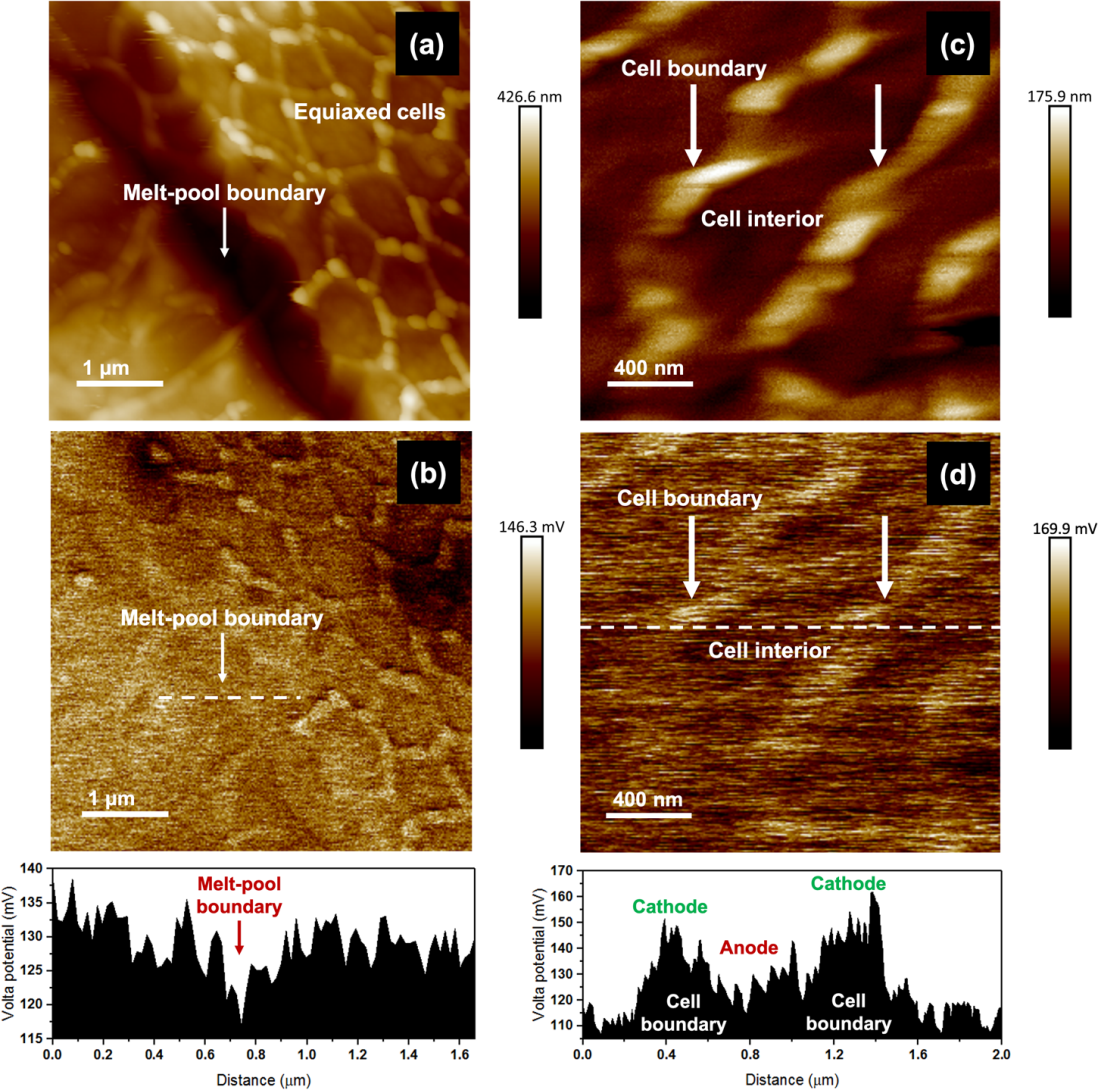
Scanning Kelvin probe force microscopy (SKPFM) maps. **a**, **c** Surface topography maps and **b**, **d** Volta potential maps and potential distribution plots of the SLM SS316L surface.

Figure 3a shows the topography map of a MPB and Fig. 3b shows the corresponding Volta potential map obtained using SKPFM. SKPFM is a high-resolution technique which can be used to predict the electrochemical reactivity of various phases or features present on the surface based on the potential measured against a Pt/Ir probe. The absolute values usually vary based on several factors such as alloying elements present, nature of the surface scanned and nature of tip/surface interaction^33^, but the potential differences between the features are reflective of their propensity to corrode. Volta potential maps of the same area (Fig. 3b) showed that the MPB was about 8–20 mV lower than the surrounding cell interiors/matrix. Therefore, these features can be expected to exhibit higher electrochemical activity, i.e., be more anodic in comparison to the matrix. The anodic nature of fusion/melt-pool boundaries have been observed in other additive manufactured alloys as well^34,35^ but not documented for AM SS316L alloys through SKPFM measurements yet. Higher magnification scans of the cellular features (topography maps in Fig. 3c and Volta potential maps in Fig. 3d) showed that the cell interiors were 20–50 mV lower than the potential of the cell boundaries which was a higher difference and more pronounced than those reported in other works^13,15^.

It can also be noted that while the compositional differences obtained from the TEM-EDS point scans did not seem significant, the enrichment of the Cr/Mo at the boundaries made a significant impact on the Volta potential difference. Since Mo is galvanically more noble than Fe (comparing their position in the electrochemical series) and Cr/Mo are the main passivating elements, the depletion of these elements in the cell interiors and MPBs makes them more prone to attack^36–38^. Electrochemically, the observation of such Volta potential differences leads to the formation of micro-galvanic cells, which causes a preferential attack of the more anodic regions when the passive film breaks down upon exposure to a corrosive environment. They may even lead to heterogeneities in the passive film formed due to local differences in concentrations of passivating elements. Therefore, even though there is a high dislocation density at the cell boundaries, the Cr/Mo enrichment had a dominating effect over the electrochemical characteristics as reported by the previous works. The influence of these heterogeneities on the localized corrosion behavior in neutral and acidic environments under ambient conditions has been well documented but whether they will play a role at elevated temperatures and in the presence of corrosive H_2_S will be discussed in the subsequent sections. There was no significant Volta potential difference observed between the nanoinclusions and the cell boundaries, which implies that they would not play any role in the electrochemical behavior on their own.

### Electrochemical impedance spectroscopy (EIS)

**Fig. 4:**
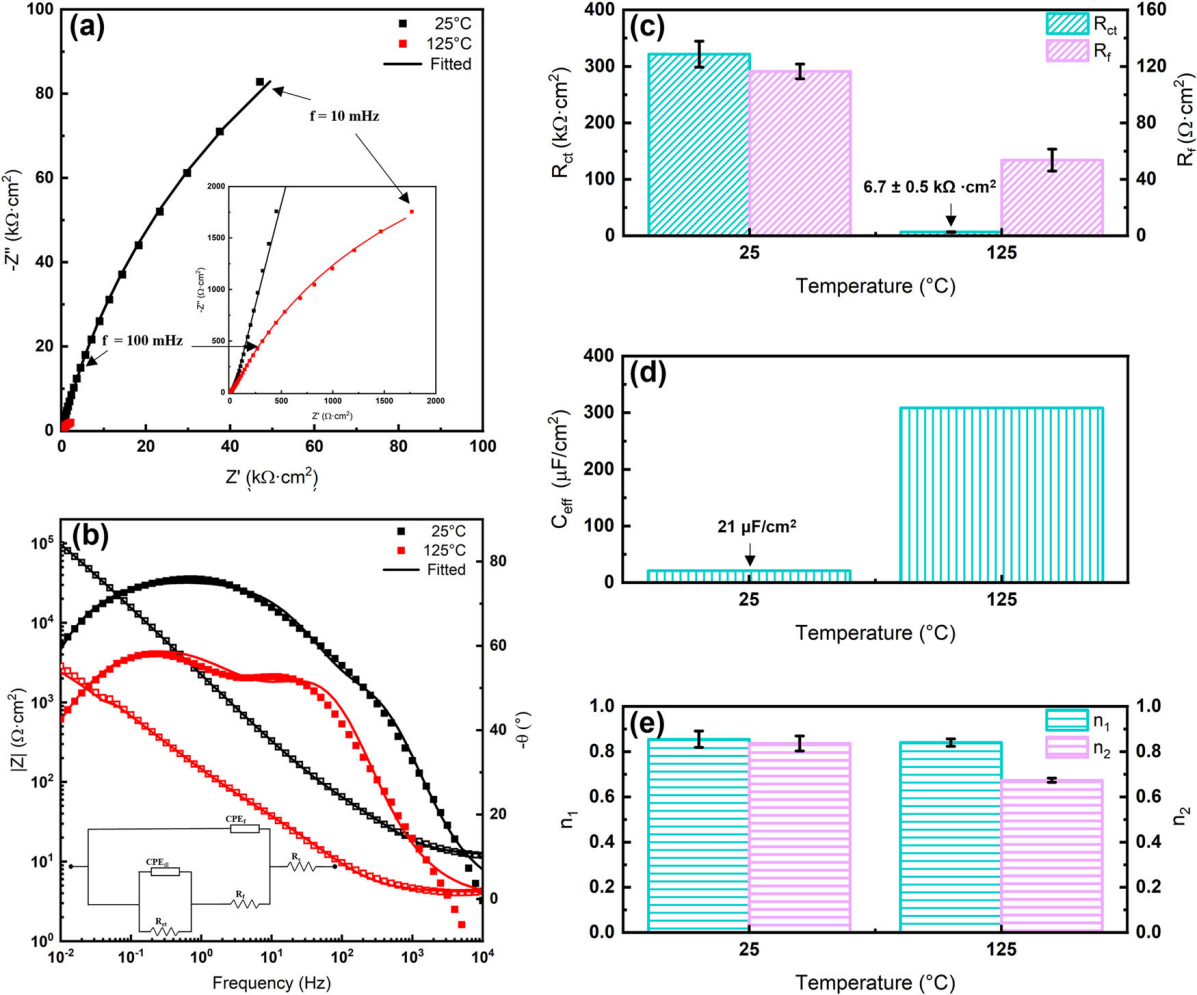
EIS results obtained after immersion of SLM SS316L in the simulated condensed natural gas brine at varying temperatures. **a** Nyquist plot; **b** Bode plot and EEC used; **c** Resistances obtained from EEC; **d** *C*_eff_ comparison between the varied temperatures; **e** *n* value comparisons.

EIS was used to observe characteristics of the passive film. The Nyquist and Bode plots are shown in Fig. 4 for SLM SS316L at 25 and 125 °C. At 125 °C, the Nyquist plots show the decreased arc diameter in comparison to 25 °C which suggests a lower corrosion performance due to possibly more charge transfer dominating processes. The Bode plots show a shift in the phase angle at the lowest frequencies (0.01 Hz) from -60° to -42° at 25 °C and 125 °C respectively, thus demonstrating the transition from a compact passive layer to a more compromised film^39^.

The proposed electrical equivalent circuit (EEC) (inset in Fig. 4b) was used to fit the experimental results, since it accounts for the passive layer formation and pit initiation^25^. A constant phase element (CPE) is used to signify a non-ideal capacitor, which is commonly used to account for uneven current distributions at the surface of the electrode^25,40^. The impedance of the CPE is given by the expression:

1 $${{\boldsymbol{Z}}}_{{\bf{CPE}}}={{\boldsymbol{Q}}}^{-{\bf{1}}}{\left({\boldsymbol{i}}{\boldsymbol{\omega }}\right)}^{-{\boldsymbol{n}}}$$

Where *Q* and *n* are parameters representing the CPE and *ω* is the radial frequency ($=2\pi f)$. The circuit is composed of the solution resistance (*R*_s_) in series with a parallel combination of the CPE_f_ and resistance (*R*_f_) of the passive film. This is in series with parallel circuit elements comprising of the CPE_dl_ of the double layer and charge transfer resistance (R_ct_).

Effective capacitance (*C*_eff_) is a parameter that quantifies the nature of the passive film providing a more physical interpretation of the system than CPE and was evaluated using the model proposed by Hsu and Mansfield^41^. The *C*_eff_ is determined by the equation:

2 $${{\boldsymbol{C}}}_{{\bf{eff}}}={{\boldsymbol{Q}}}^{{{\bf{1}}}/{{\boldsymbol{n}}}}{{\boldsymbol{R}}}_{{\boldsymbol{f}}}^{({{\bf{1}}-{\boldsymbol{n}})}/{{\boldsymbol{n}}}}$$

The higher tendency to form metal sulfides in the passive film at elevated temperatures is accounted for by the decrease in *R*_*f*_ and *R*_ct_ which implies an increased corrosion rate, and the increase in *C*_eff_ (see Fig. 4c, d) and Table 1). This has been attributed to the enhanced diffusion kinetics of S through the film and higher S adsorption^25^. The heterogeneity of the passive film and double layer are characterized by *n*_*1*_ and *n*_*2*_ respectively, where *n* = 1 represents a pure capacitor and *n* = 0 a pure resistor^1^. At 125 °C, the dissolution rate of the alloy is higher in the H_2_S-CO_2_ containing system due to the increased defective nature of the passive film from the enhanced sulfide adsorption^1,25^. The ingress of the sulfides to the inner layer of the film likely contributes to the decrease in *n*_*2*_ (Fig. 4(e) and Table 1) which will be discussed in the XPS section.

**Table 1:** Calculated equivalent circuit parameters in the sour simulated brine solution

*T*	*R*_s_	*Q*_f_	*n*_1_	*R*_f_	*Q*_dl_	*n*_2_	*R*_ct_	*C*_eff_	*χ*^2^
(°C)	(Ω cm^2^)	(S^−1^ cm^−2^ s^n^)		(Ω cm^2^)	(S^−1^ cm^−2^ s^n^)		(kΩ cm^2^)	(μF cm^−2^)	
25	12.1	$5.04\times \,{10}^{-5}$	0.85	116	$4.49\,\times \,{10}^{-5}$	0.84	322	21.0	$1.01\,\times \,{10}^{-3}$
125	4.15	$5.94\,\times \,{10}^{-4}$	0.84	53.7	$1.47\,\times \,{10}^{-3}$	0.67	6.73	309	$7.32\,\times \,{10}^{-3}$

Reference ^1^ studied the performance of wrought SS316L in acidic environments with varying concentrations of H_2_S at 60 °C. The EIS showed a single capacitive loop simulated by a Randles circuit indicating the sample continuously corroded due to the porous layer of FeS_2_ and MoS_2_ providing negligible protection. Reference ^25^ evaluated the performance of wrought SS316L exposed to 0.1 MPa of H_2_S at 30 °C and 130 °C, and demonstrated greater passivity than the aforementioned study by exhibiting more negative phase angles of around −40° and −20° respectively. While the study failed to account for the changes in the H_2_S concentration in the aqueous phase due to changes in the solubility at elevated temperatures, this was maintained constant in the present study with a higher salt concentration being used. The corrosion resistance of the SLM samples was higher despite more extreme testing parameters, exhibiting more negative phase angles (-40 to -60°) in the lower frequencies, higher *R*_*f*_ and *R*_*ct*_, and *n* values closer to 1.

### Mott–Schottky

Comparisons of the defect densities and semiconductive properties of the passive film formed at 25 and 125 °C were made using Mott–Schottky analysis. The Mott–Schottky relation shows the influence of the potential on the capacitance of the space-charge layer (*C*_*sc*_) under the depletion condition. The equation used to determine the defect densities contributed by p-type n-type semiconductors is:

3 $$\frac{{\bf{1}}}{{{\boldsymbol{C}}}_{{\boldsymbol{sc}}}^{{\bf{2}}}}=\pm \frac{{\bf{2}}}{{\boldsymbol{\varepsilon }}{{\boldsymbol{\varepsilon }}}_{{\bf{0}}}{\boldsymbol{eN}}}\left({\boldsymbol{E}}-{{\boldsymbol{E}}}_{{\boldsymbol{FB}}}-\frac{{\boldsymbol{kT}}}{{\boldsymbol{e}}}\right)$$

where, ε is the dielectric constant of the passive film (15.6), ε_0_ is the vacuum permittivity (8.854 × 10^−14 ^F cm^−1^), *e* is the electron charge (1.602 × 10^−19 ^C), $N$ is the defect density, $k$ is the Boltzmann constant (1.38 × 10^−23 ^J K^−1^), T is the absolute temperature, $E$ is the applied potential (V_SCE_), and ${E}_{{FB}}$ is the flatband potential (V_SCE_).

**Fig. 5:**
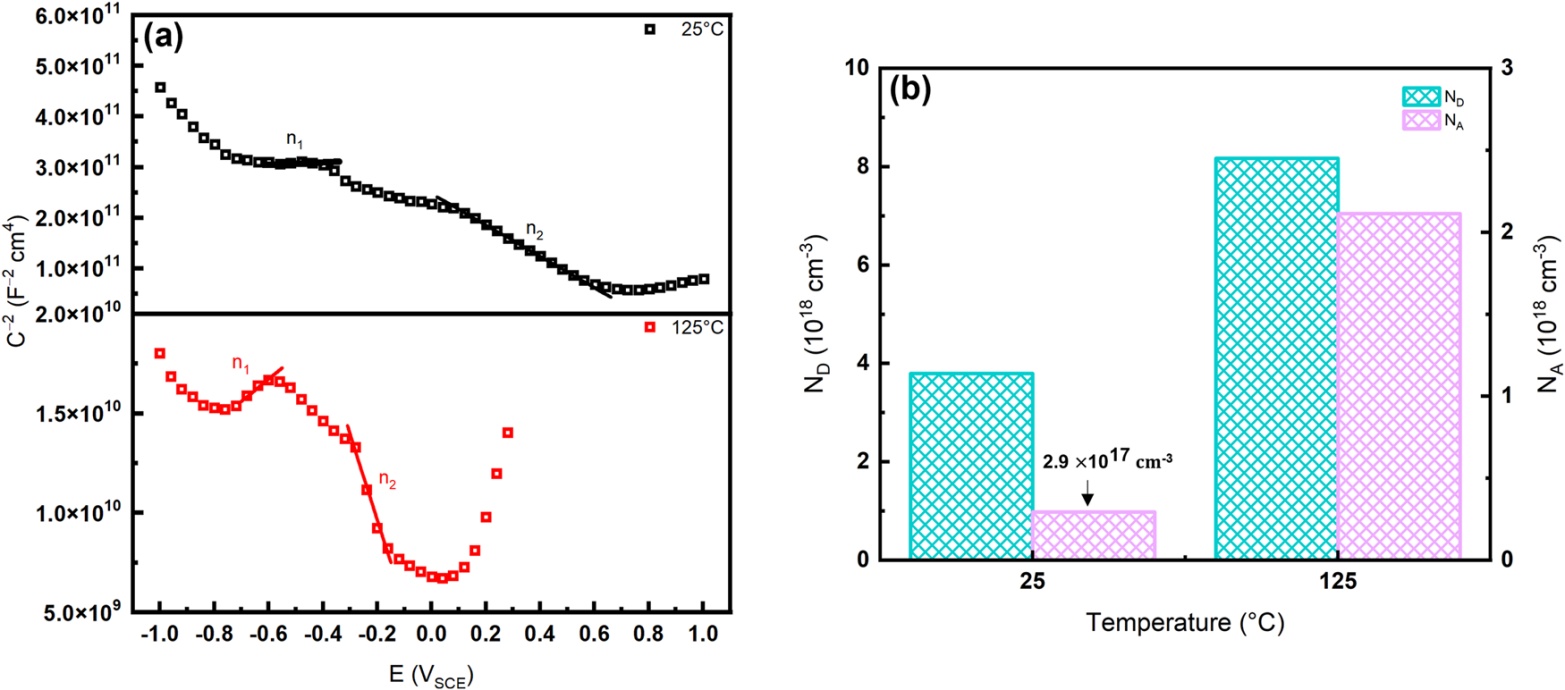
Mott-Schottky results. **a** Mott–Schottky plots and **b** calculated defect densities of passive films formed on SLM SS316L in the simulated sour brine.

Figure 5(a) shows the Mott–Schottky plots of the passive films generated on SLM SS316L at 25 and 125 °C. The graphs are shown to have positive and negative slopes separated by a plateau region where ${E}_{{FB}}$ is found. Thus, the passive film of SLM SS316L exhibited both p-type and n-type semiconductor behavior at OCP due to the presence of the p-n heterojunction^39^. Passive films formed on wrought SS316L and duplex steel in H_2_S-containing environments have generally been found to exhibit the electronic structure of the p-n heterojunction regardless of the temperature^25,39^. At potentials lower than ${E}_{{FB}}$, a positive slope is shown at both temperatures indicating n-type semiconductor behavior, where the charge carriers are associated with oxygen vacancies and cation interstitials. Compounds including Fe_2_O_3_, FeOOH, MoO_3_, and metal sulfides exhibit n-type behavior^25,42,43^. At potentials higher than ${E}_{{FB}}$, a negative slope implies p-type semiconductor behavior analogous to cation vacancies^2,39,44^. Components in the passive film exhibiting p-type semiconductive behavior include Cr_2_O_3,_ Fe_3_O_4_, NiO, and Ni(OH)_2_^25,42^. Reference ^45^ determined the presence of the p-n heterojunction was a consequence of the formation of a bilayer structure, where constituents with differing semiconductor behaviors were enriched in the inner and outer layers.

The penetration of Cl^−^ ions under the influence of an electric field (~10^6 ^V cm^−1^) was first discussed by ref. ^46^. This uptake leads to an increase in the ionic conductivity along the penetration path, leading to an auto-catalytic process. The point defect model (PDM) expanded this idea to develop a framework to discern passive film growth along with film breakdown and pit initiation with respect to point defects generated in the oxide layer^44,47,48^. The occupation of Cl^−^ ions in oxygen vacancy sites at the film/solution compensates for the loss of oxygen vacancies by generating cation vacancy and oxygen vacancy pairs through a Schottky-pair reaction or via cation abstraction^44,48–50^. The cation vacancies generated move to the metal/film interface, where they are annihilated by cation injection into the film from the substrate. Condensation of the cation vacancies along the metal/film interface occurs if the flux of cation vacancies exceeds the annihilation rate^51^. As a result, local separation induces stresses within the film and its subsequent breakdown.

The defect densities were calculated from the linear fits of the Mott–Schottky plots (n_1_ and n_2_) (Fig. 5b). With increasing temperature, there is an observed increase in the electron donor density (*N*_*D*_) from n-type semiconductors and the hole acceptor density (*N*_*A*_) from p-type semiconductors. The increase in temperature would decrease the thickness of the double layer, inducing a high electric field to increase the reaction kinetics. As a consequence, this would lead to an increase in the exchange rate, where the cation and anion elements in the passive film were susceptible to appear in the wrong positions, leading to higher defect densities. In comparison to wrought SS316L exposed to sour environments at ambient conditions^25^, the defect densities were found to be significantly lower for both temperatures (~10^18 ^cm^−3^) than the wrought sample, despite being exposed to higher pressures (bal. CO_2_) while maintained at the same pH of 4. These values were also significantly lower (by a factor of 1000) than wrought SS316L bubbled with H_2_S and CO_2_^52^.

The PDM suggests that cation vacancies tend to accumulate at sites where inclusions (e.g., MnS), precipitates (Cr_23_C_6_), and dislocations are prominent. This phenomenon is attributed to the high lattice disorder along the intersection between these regions and the barrier layer, which induces higher cation vacancy diffusivities^49,53^. Consequently, pits are preferentially formed along these regions. However, the competing effects between the high dislocation densities along the cell boundaries and the cathodic nature of boundary, as indicated by the SKPFM, complicates the determination of which effect dominates. Further discussion on preferential sites for attack will be carried out in the subsequent sections.

### X-ray photoelectron spectroscopy (XPS) and scanning electron microscopy (SEM) after tests

After the 24 h exposure of the SS316L samples at 25 and 125 °C, XPS analysis was done on the surface to reveal the oxide composition. The survey XPS spectra (not included) exhibited signals from Fe, Cr, Mo, Ni, O, S, and C and Supplementary Table 1 shows the amounts of the elements calculated from the survey scans after 10, 20, and 50 min of sputtering. With increase in temperature, there was a 47% decrease in the Fe content (from 10.27 to 5.39 at%), a decrease of 23% in the Cr content (from 9.79 to 7.54 at%), and a substantial increase of S of 345% (from 5.27 to 23.47 at%). The reduction in Fe and Cr is possibly due to the reduction in protective oxides and an increase in sulfide products at 125 °C. Supplementary Table 2 shows the peak identification parameters as well as the at% of each deconvolution after 10 min of sputtering the surface.

**Fig. 6:**
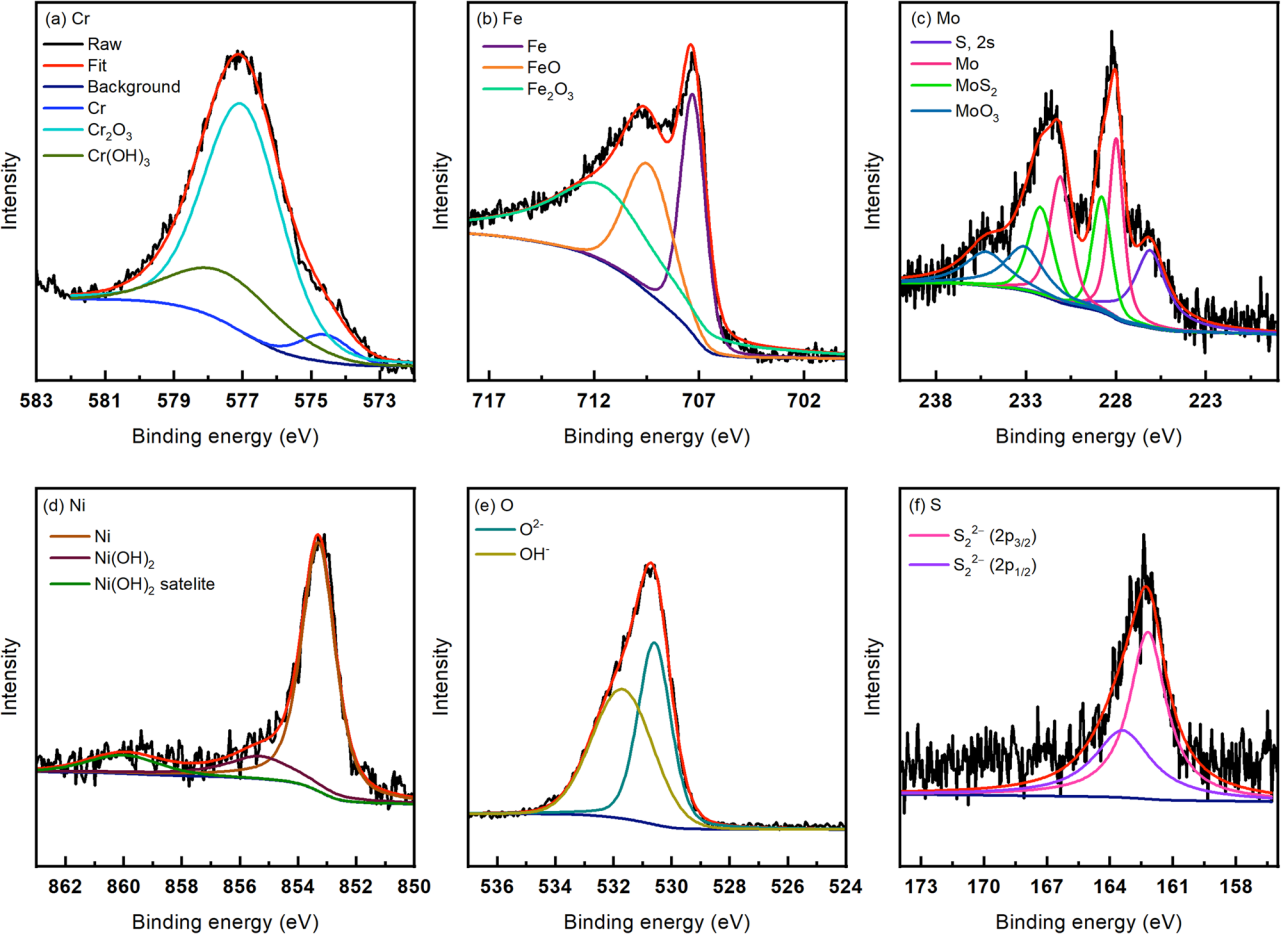
High-resolution XPS spectra (after 10 minutes of sputtering) after 24 h at 25 °C. **a** Cr, **b** Fe, **c** Mo, **d** Ni, **e** O, and **f** S.

**Fig. 7:**
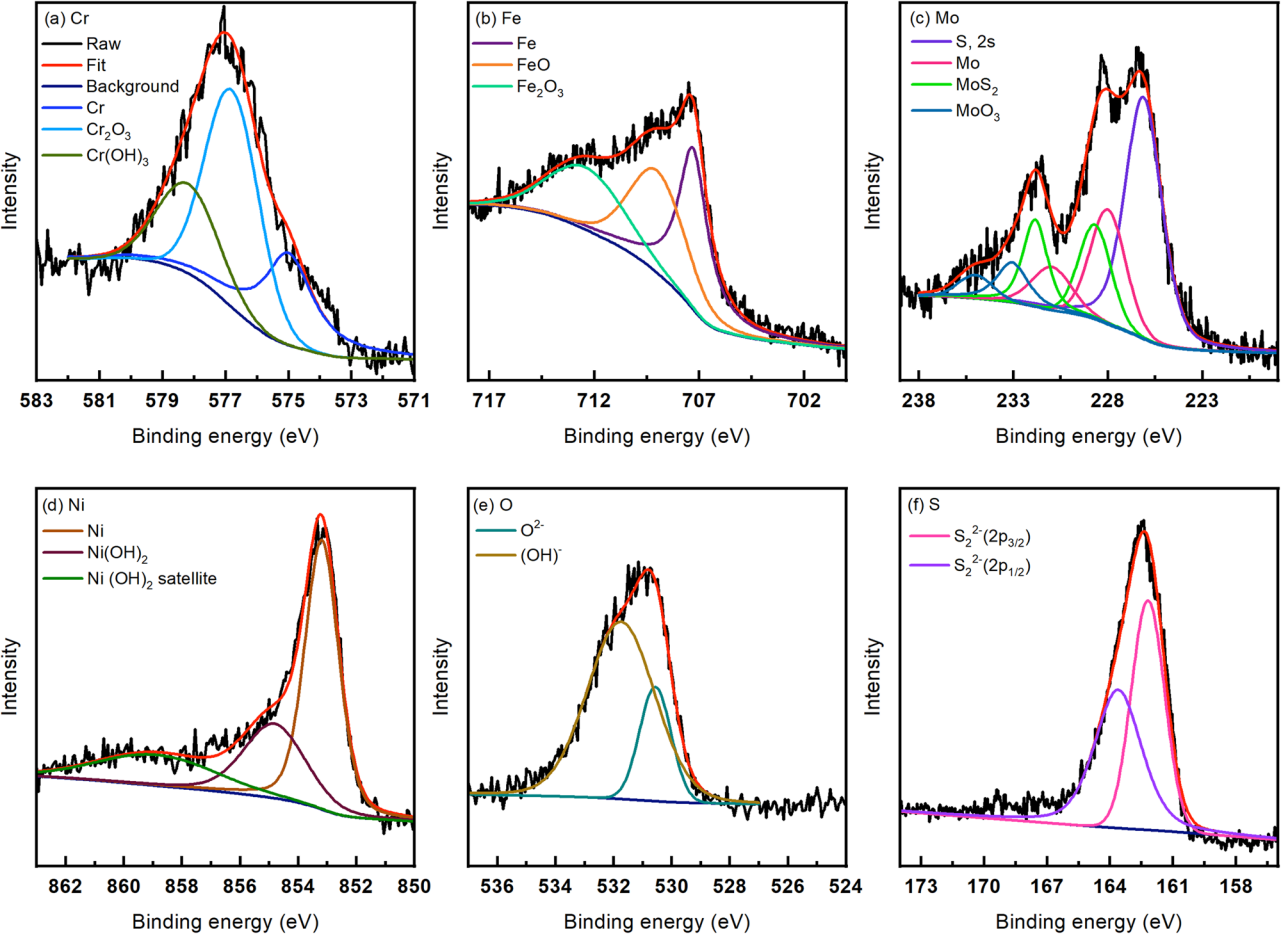
High-resolution XPS spectra (after 10 min of sputtering) after 24 h at 125 °C. **a** Cr, **b** Fe, **c** Mo, **d** Ni, **e** O, and **f** S.

**Fig. 8:**
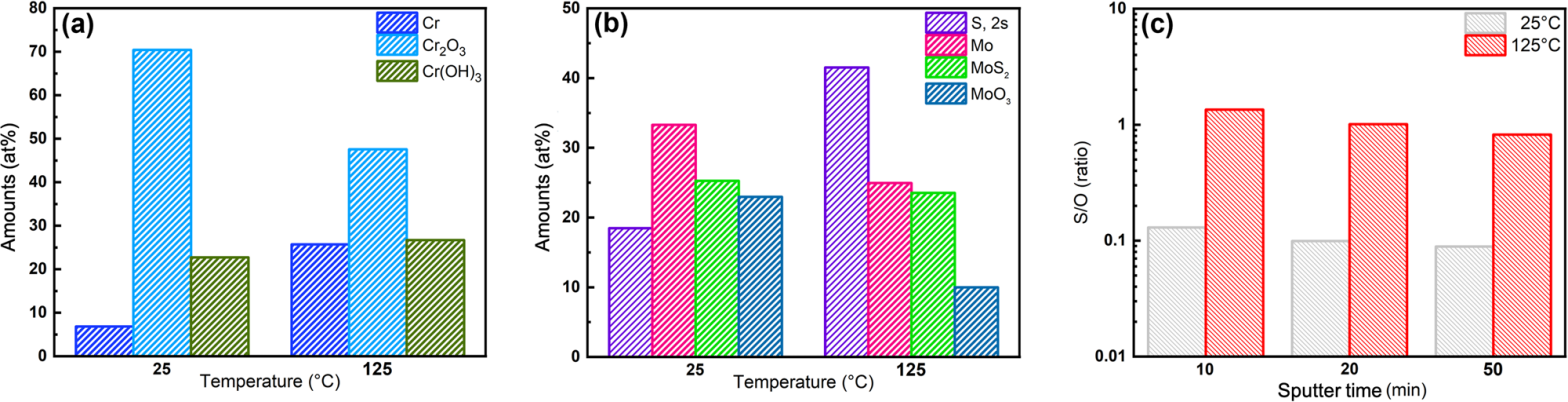
XPS quantitative analysis. Amount of **a** Cr components, **b** Mo components, and **c** S/O ratio obtained from the XPS spectra at the two test temperatures.

Figures 6 and 7 show the high-resolution XPS spectra at 25 and 125 °C after sputtering the surface for 10 min. The at% of Cr and Mo deconvolutions at the two tested temperatures are shown in Fig. 8a, b respectively. It can observed that while the Cr(OH)_3_ content only slightly increased from 25 to 125 °C, there is a substantial decrease in protective Cr_2_O_3_ content (from 70.4 to 47.55 at%). In pure CO_2_ containing environments, refs. ^54,55^ claimed a reduction in Cr_2_O_3_ is apparent, along with an increase in Cr(OH)_3_. The presence of Cr(OH)_3_ has been reported to increase defect concentration in the passive film that contributes to higher corrosion kinetics^56^. However, in CO_2_ and H_2_S containing environments, ref. ^57^ attributed the Cr_2_O_3_ enrichment in the passive film to the presence of CO_2_. Thus, CO_2_ is shown to stabilize the presence of oxides in the layer and negate the effects of S uptake in comparison to environments without CO_2_. This could possibly be the reason why passivity is still observed at elevated temperatures even though the film formed is more defective and more prone to breakdown. Upon increasing temperature, it can be observed from the Mo 3d peak that the S 2 s peak inside of it increased (from 18.48 to 41.53 at %), coinciding with the overall increase in S. Additionally, the Mo oxide percentages substantially decreased at 125 °C (Fig. 8 (b)) agreeing with the lower passivity from the EIS results. Furthermore, there is a decrease in the protective oxide percentage of both Fe_2_O_3_ and Cr_2_O_3_, coinciding with the higher susceptibility to film breakdown seen by the decrease in the *R*_*f*_.

There is also an increase in the Ni(OH)_2_ content, where less Ni metal is seen (decrease from 73.11 to 53.95 at%) and more overall Ni presence (63% increase) at 125 °C. It is difficult to differentiate elemental Ni from Ni sulfides and therefore, there could be some contribution of sulfides to this peak as well. The S/O peak ratios (Fig. 8c) after 10, 20, and 50 min of sputtering were 0.13, 0.099, and 0.089 at 25 °C while at 125 °C they were 1.35, 1.01 and 0.82. Therefore, this was in agreement with the EIS results that expected higher sulfur ingress to the inner layer of the film at 125 °C than 25 °C. The higher amount of S in the outer layer of the film at higher temperatures has been attributed to higher adsorption of S^25^. The sulfides generated in the passive film were thus found to be Mo sulfides and possibly Ni sulfides that were difficult to deconvolute from the XPS spectra but were identified through EDS maps as will be discussed in the subsequent section.

While the XPS results provide a general idea on the nature of the passive film formed, there could be many local differences in the passive film formed due to the chemical segregations of passivating elements such as Cr and Mo. Reference ^58^ through TEM imaging showed that the inner Cr oxide rich film nucleated at the Mn/Si nanoinclusions and the cell boundaries which were enriched in Cr/Mo in stress-relieved laser powder bed fusion (LPBF) SS316L samples when exposed to neutral solutions. Additionally, the passive film was found to be heterogenous with increased thickness at the boundaries. In comparison, the wrought SS316L showed a uniform passive film throughout the imaged surface. The high cell boundary density and dislocation networks have also been reported by several works to be the nucleating sites for the oxide film and reason for formation of thick passive films in additively manufactured SS316L^9,13^. Whether the same was the case in the alloy and acidic system focused in this study can only be concluded upon using TEM and higher-resolution techniques but has not been performed since this was not the aim of the work. However, similar heterogeneities can be expected based on the localized corrosion behavior exhibited by the SLM SS316L as will be discussed in the subsequent section.

**Fig. 9:**
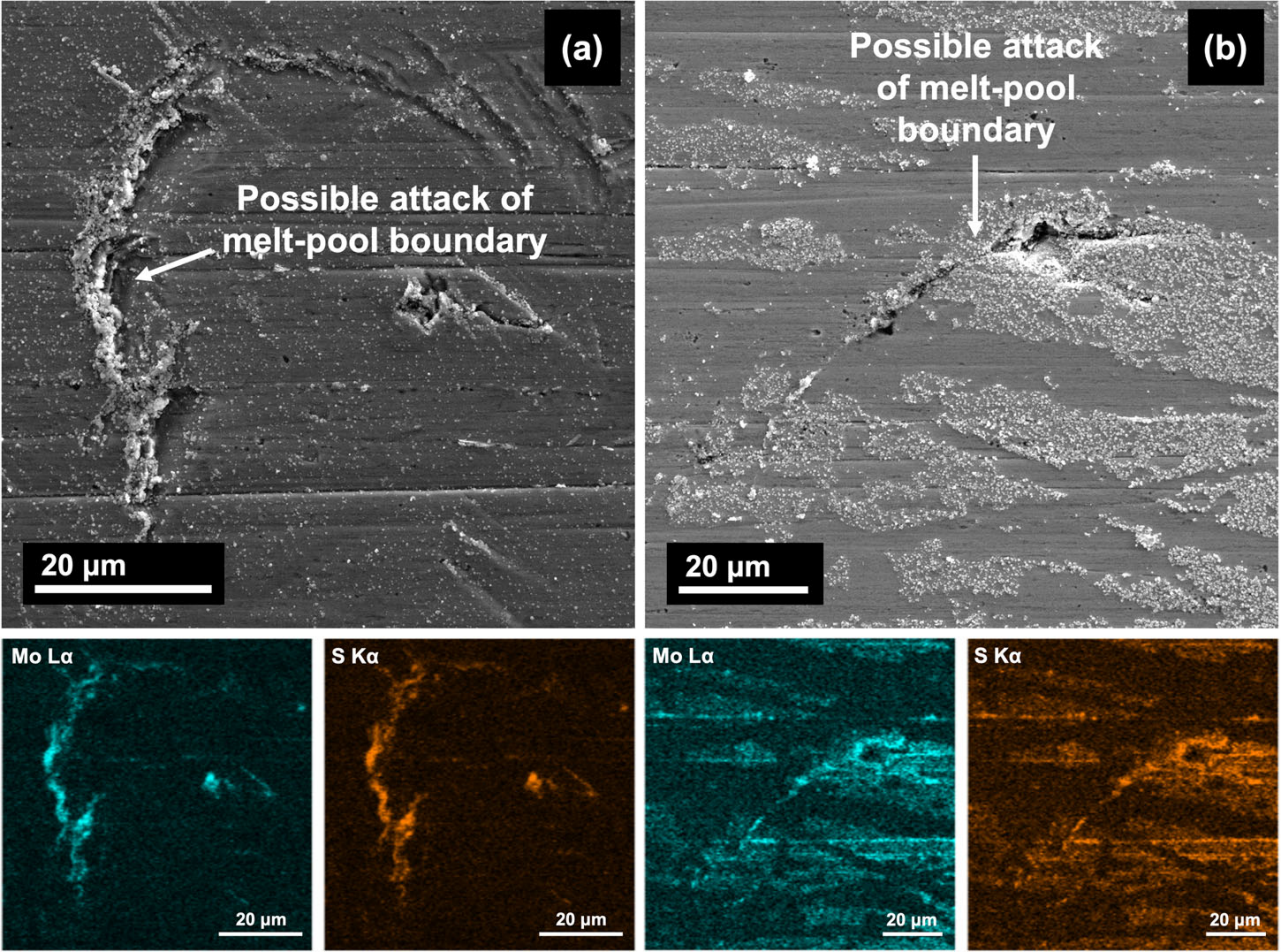
SLM SS316L after one day of immersion at 125 °C. **a**, **b** SEM images showing possible regions of attack at the melt-pool boundary and the formation of molybdenum sulfides at these regions.

After one day of exposure at 25 °C, the surfaces showed no obvious signs of attack and hence the images have not been included in the work. At 125 °C, the exposed surface was mostly intact but certain features were found to be present at some areas (shown in Fig. 9). These appeared to be regions that showed some signs of attack and resembled the arcs of the MPBs considering the scale of these features. EDS mapping of these areas also showed the formation of Mo sulfides in these regions of attack. Taking into account the lower Volta potential at the MPBs and the scale of these localized regions of attack, it can be concluded that these were the preferential regions of initial attack. The presence of sulfides in these regions also corroborated the hypothesis from the EIS section as well as results from XPS that stated that higher sulfide formation when tested at 125 °C reduced the stability of the passive film and made the SLM SS316L more prone to earlier onset of localized corrosion.

**Fig. 10:**
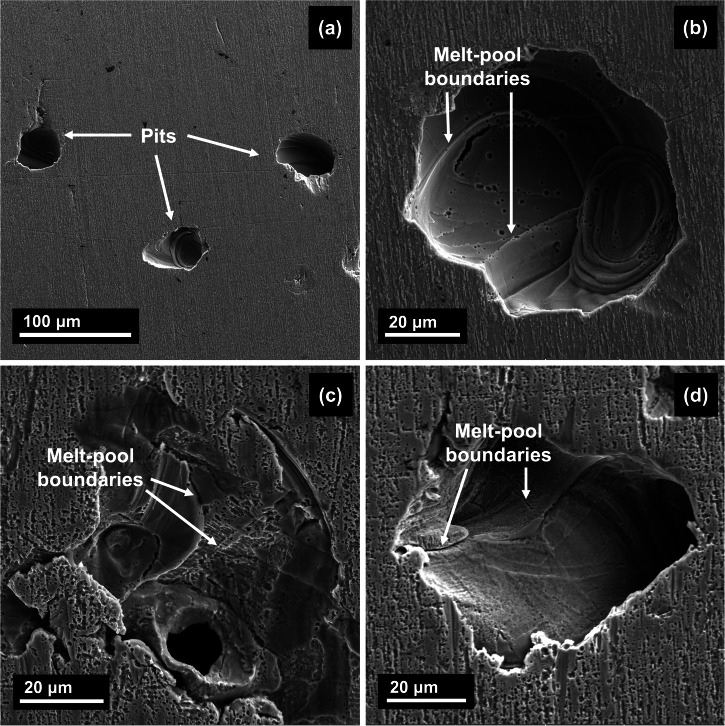
SLM SS316L after one week of exposure at 25 °C. SEM imaging of the sample showing **a** pits formed on the surface and **b**–**d** evidence of attack at melt-pool boundaries within the pit interiors.

**Fig. 11:**
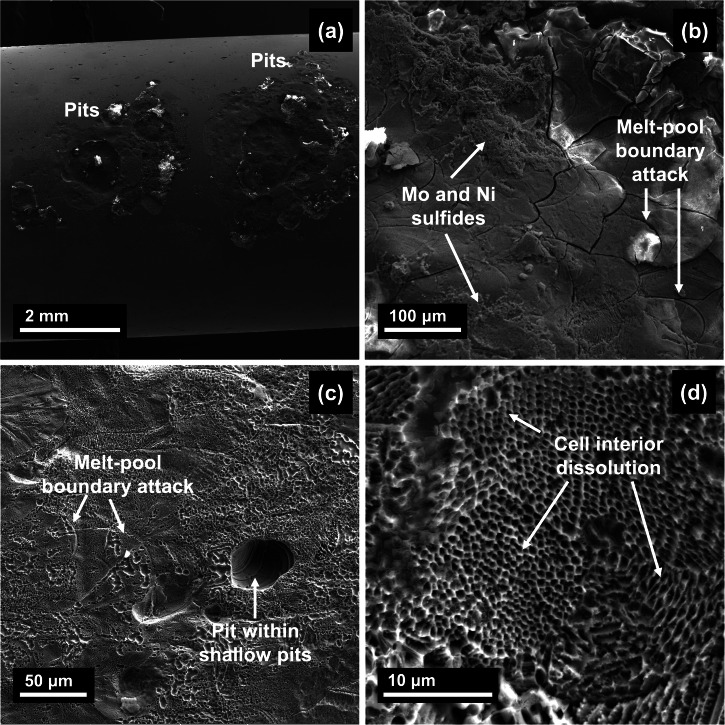
SLM SS316L after one week of exposure at 125 °C. **a** Large pits that covered most of the surface, **b**, **c** attack at melt-pool boundaries within the pits and **d** evidence of cell-interior dissolution.

After one week of exposure at 25 °C, the surface was covered with pits that ranged from 30–60 μm (Fig. 10a). Upon looking into magnified images of the pits, there was evidence of attack at the MPBs in the underlying layers to the attacked surface (Fig. 10b–d). This preferential attack is analogous to intergranular corrosion of regions depleted of Cr/Mo^16,18^. However, at 125 °C a more severe attack was observed where the pits were wider by a whole order of magnitude with them ranging from 50 μm to 3 mm (Fig. 11a). Due to the earlier breakdown of the film, in the same one week of exposure, there is more time as well as accelerated kinetics due to higher temperatures that allows all the pits formed to coalesce and form these wider areas of attack. Upon looking into the corroded interiors of the pits, there is further evidence of attack at the MPBs (Fig. 11b, c). Corrosion products were also found at certain regions within the pit (indicated in Fig. 11(b)) which were confirmed to be Ni and Mo sulfides using EDS (maps presented in Supplementary Fig. 2). The formation of such corrosion products has been reported in martensitic stainless steels by ref. ^59^ as well. Looking into the corroded regions near the MPBs, the occurrence of cell-interior dissolution was observed where the cell boundaries appeared to be intact.

**Fig. 12:**
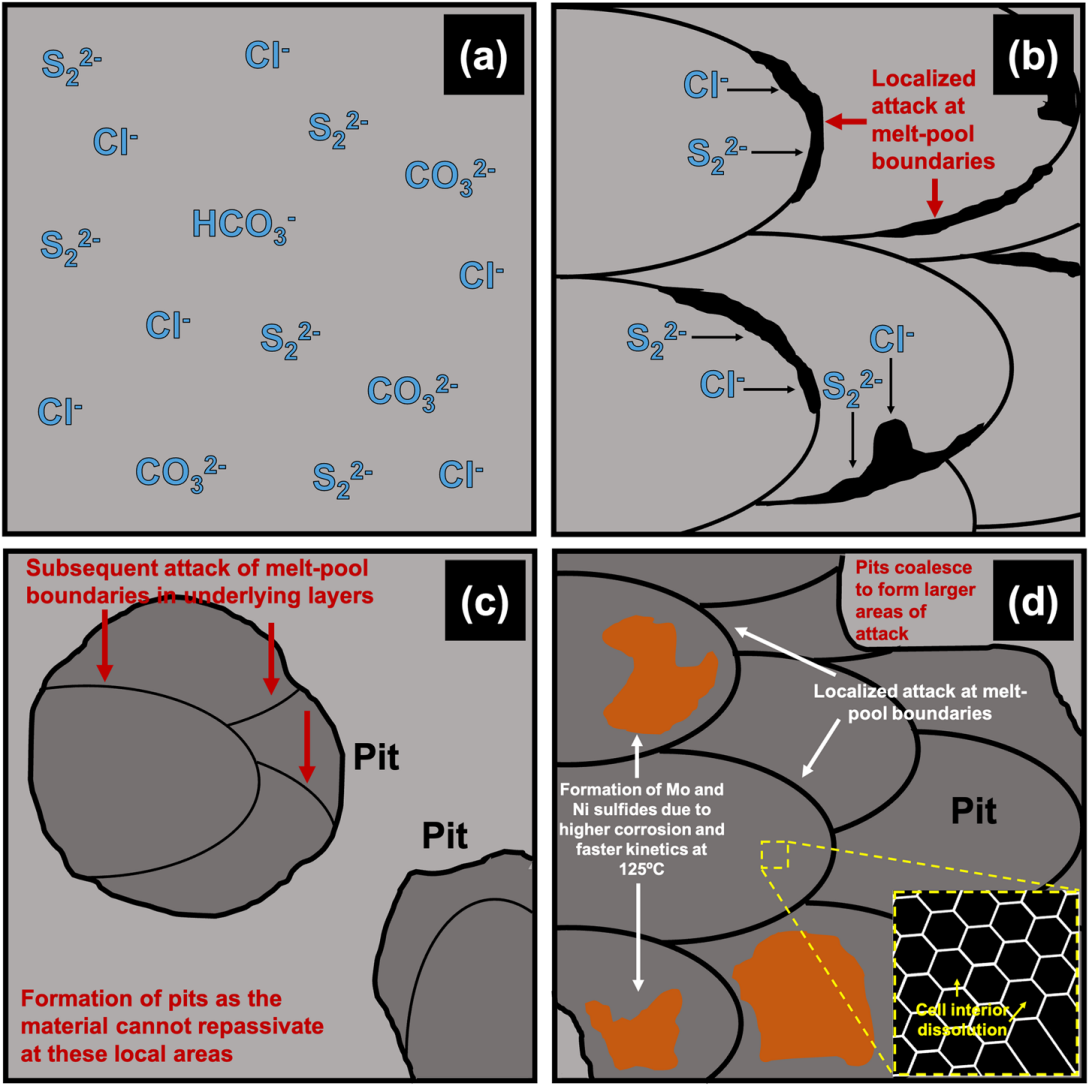
Proposed mechanism of localized attack on SLM SS316L. **a** Environment containing CO_2_ and H_2_S which creates acidic conditions and deteriorates the stability of the passive film and Cl^−^ ions responsible for localized attack; **b** Localized attack at MPBs due to lower Cr/Mo at these regions leading to pit initiation; **c** Formation of pits due to the dissolution of exposed surface which causes subsequent attack of MPBs in the underlying layers (as observed at 25 °C); **d** At higher temperatures (at 125 °C), these pits coalesced to form larger areas of attack across the surface that also showed MPB attack as well dissolution of cell interiors in the regions surrounding these areas.

At temperatures as high as 125 °C and in the acidic environments tested, severe corrosive attack is expected to occur before composition and potential differences in the micro- and nano-scale can play any role. However, these differences have been found to make an overall impact on the localized corrosion behavior once the passive film broke down. Therefore, lower Cr/Mo at the MPBs possibly led to the passive film being more susceptible to breakdown at these regions. As the passive film broke down due to the acidic environments caused by the CO_2_, they become local anodes (due to them having a lower Volta potential) and start to locally dissolve. Once the exposed top surface dissolves, the underlying areas are exposed to the environment, and subsequent attack of these features in these layers occurred. At higher temperatures, due to enhanced kinetics and the less protective nature of the passive film, the small pits formed possibly coalesced to form large areas of attack across the surface. There was still evidence of attack at MPBs but also dissolution of the cell interiors in the regions surrounding the MPBs. Therefore, once the film breaks down, the areas that served as local anodes due to the depletion of passivating elements such as Cr/Mo were found to be the preferential sites of attack. This has been summarized in the form of a schematic in Fig. 12.

The contradiction between works reporting whether the cell boundary, inclusion, MPB or cell interior was the region most susceptible to attack could be due to different printing parameters causing a shift in whether the dislocation density, nature of inclusions or the Cr/Mo micro-segregation played a significant role. Additionally, the available literature discussing the mechanisms of attack utilized cyclic polarization with varying parameters to carry out the localized corrosion study which can vary the degree of attack observed on the surface after the tests. In this work, the results observed were all a consequence of immersion under open circuit potential conditions and not due to any polarization which could be more reflective of which features were naturally susceptible to attack under practical service conditions. The nature of the test electrolyte could also be a contributing factor as to which parameter influenced the electrochemical behavior. Therefore, a deeper understanding of the microstructural features developed upon additive manufacturing and how the environment interacted with the samples can help understand the competitive effect between the various heterogeneities to determine which parameter contributed to preferential sites of attack.

To summarize the results obtained in this study, TEM characterization revealed higher dislocation density at cell boundaries which should ideally contribute to them being more prone to selective attack. However, Cr/Mo enrichment at these regions caused a higher Volta potential at boundaries than interiors which made them cathodic and less prone to localized attack. Additionally, a depletion of Cr/Mo was observed at the melt-pool boundaries (MPBs) which also reflected in their Volta potential being lower than that of the matrix. Therefore electrochemically, cell interiors and MPBs can be said to be more electrochemically active and would serve as local anodes. The EIS and Mott–Schottky results showed a more defective passive film with a decrease in corrosion resistance at 125 °C than 25 °C due to increased ingress of sulfide species into the inner layer of the film forming more metal sulfides than protective oxides/hydroxides. XPS results showed a reduction in protective Cr_2_O_3_ and Mo oxides with an increase in Mo and possible Ni sulfides at 125 °C than 25 °C. This possibly led to earlier breakdown of the film which caused severe pitting corrosion to ensue. While the SLM SS316L did exhibit significant passivity when exposed to elevated temperatures and environments containing H_2_S, the local chemical heterogeneities compromised the stability of the film. The printing parameters would therefore have to be engineered carefully to avoid such chemical segregations to possibly improve the corrosion performance of AM SS316L.

## Methods

### Sample preparation

Cylindrical SLM SS316L samples (height 46 mm, diameter 6.3 mm) were manufactured using an EOS M 100 DMLS printer with Powder Alloy Corporation UNS31603 powders of size (45 ± 15 μm) on a SS304L substrate. A laser power of 170 W, scan orientation of 90°, scan speed of 800 mm/s, layer height of 20 μm, and hatch spacing of 120 μm were selected as the printing parameters. For the electrochemical tests, the as-printed cylindrical samples were polished up to 1200 grit SiC emery paper to remove any surface roughness, drilled and threaded at one end to fit the ports in the autoclave. For the X-ray photoelectron spectroscopy (XPS) immersion, polished samples were vertically sliced and holes drilled through them in order to make them suitable for suspension in the autoclave with a wire. For initial microstructural characterization, the as-printed materials were sliced vertically to obtain samples exposing the area parallel to the build direction and were ground up to a 1200 grit SiC emery paper. They were fine polished in a napped cloth using a 9 μm, 3 μm, and 1 μm diamond suspension solution and finishing polishing was performed using a 0.04 μm SiO_2_ suspension.

### Initial microstructural characterization

For transmission electron microscopy, focused-ion beam (FIB) lift out-samples were prepared from the polished samples using a FEI Helios NanoLab 650 and imaging was performed using a JEOL 2800 scanning transmission electron microscope (STEM). Additional microstructural characterization was carried out on the samples etched with aqua regia (3:1 HCl: HNO_3_) using an optical microscope (Nikon Eclipse MA 100 inverted microscope) and a Tescan FERA3 FIB scanning electron microscope (SEM) with an accelerating voltage of 15 kV at a working distance of 9 mm. Surface topography and Volta potential maps were obtained via scanning Kelvin probe force microscopy (SKPFM) performed in a Bruker Dimension Icon atomic force microscope (AFM). A Bruker SCM-PIT-V2 probe was used with a tapping/lift sequence (scan rate = 0.5 Hz, lift scan height = 80 nm) on the etched surface.

### Experimental design

**Fig. 13:**
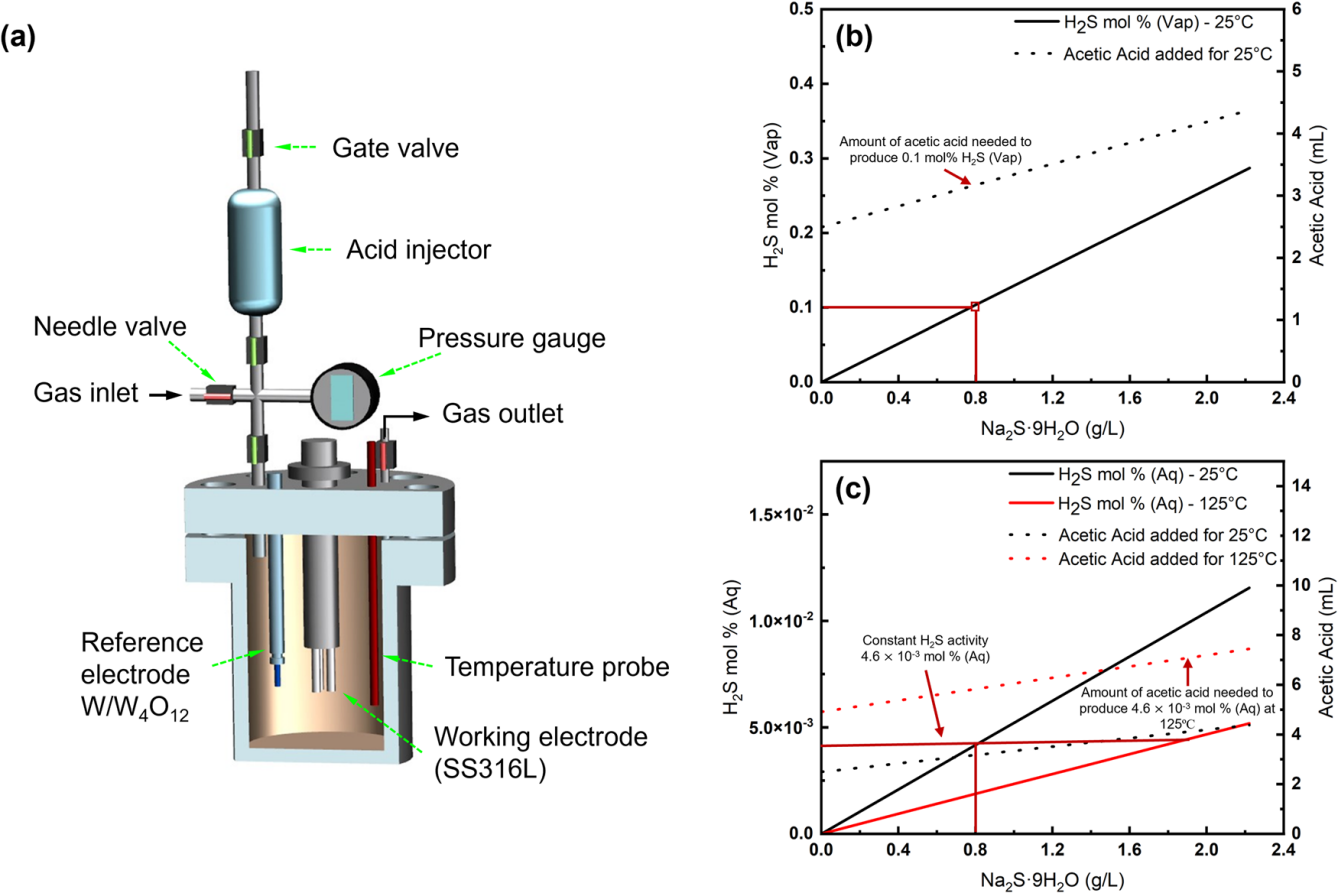
Testing methodology. **a** Schematic of autoclave used for testing, where the needle valves regulate the gas flow and the gate valves separate the acid injector and autoclave brine for individual purging; **b** Initial procedure to obtain respective Na_2_S·9H_2_O and acetic acid amounts to achieve 0.1 mol% H_2_S in the vapor phase at 25 °C; **c** Subsequent procedure to obtain amount of Na_2_S·9H_2_O and acetic acid to maintain constant H_2_S activity in the aqueous phase at 125 °C as in 25 °C.

Electrochemical tests were performed in a 1-L Hastelloy C-276 autoclave (schematic shown in Fig. 13(a)) filled with 450 mL of 3.5 wt% NaCl solution with 4.0 g/L sodium acetate and Na_2_S·9H_2_O. H_2_S was generated by the addition of acetic acid into the brine. The pH was maintained at 4.0 in the CO_2_ pressurized system by using an acetate buffer (composition obtained from the OLI survey scan). A basis of 0.1 mol% H_2_S in the gas phase at 25 °C was used with the procedure to calculate initial Na_2_S·9H_2_O and acetic acid amounts shown in Fig. 13b. The procedure used to maintain constant aqueous phase H_2_S activity at both temperatures is shown in Fig. 13c. Based on these calculations, 0.8 g/L Na_2_S·9H_2_O and 3.1 mL of acetic acid satisfied the conditions for 25 °C, while the same was consistent with 2.0 g/L Na_2_S·9H_2_O and 7.3 mL of acetic acid at 125 °C. The acid was present in an isolated acid injector system. The autoclave alone was purged with N_2_ for 2 hours first after which it was isolated with the acid being purged next for 30 min. The outlet lines of both the autoclave and acid were connected to an NaOH scrubber to deaerate the system. After oxygen removal, the acid was then injected. The autoclave was pressurized to 3500 kPa using CO_2_. The temperature was controlled using a 1400 W heating jacket. The experiments were carried out by maintaining the autoclave at constant temperatures of 25 and 125 °C.

### Electrochemical tests

A three-electrode configuration was used for the electrochemical testing. The polished and threaded sample (working electrode) was placed on a port inside the autoclave. The body of the autoclave was used as the counter electrode, and a W/W_4_O_12_ reference electrode was employed with the values obtained converted to a saturated calomel electrode (SCE) basis through the relation:

4 $${{\boldsymbol{E}}}_{{\boldsymbol{SCE}}}={{\boldsymbol{E}}}_{{\boldsymbol{W}}/{{\boldsymbol{W}}}_{{\bf{4}}}{{\boldsymbol{O}}}_{{\bf{12}}}}^{^\circ }-\frac{{\bf{2.303}}{\boldsymbol{RT}}}{{\boldsymbol{F}}}\text{pH}-\frac{{\bf{2.303}}{\boldsymbol{RT}}}{{\bf{2}}{\boldsymbol{F}}}{\bf{log }}\left({{\boldsymbol{a}}}_{{{\boldsymbol{H}}}_{{\bf{2}}}{\boldsymbol{O}}}\right)-{\bf{0.242}}\text{V}$$

where ${E}_{W/{W}_{4}{O}_{12}}^{^\circ }$ is the standard cell potential of the W/W_4_O_12_ electrode, $R$ is the universal gas constant (=8.314 J mol^−1^ K^−1^), $T$ is the absolute temperature (in K), $F$ is Faraday’s constant (=96,485 C mol^−1^), and ${a}_{{H}_{2}O}$ is the activity of water.

After acid injection and subsequent CO_2_ pressurization, the specimens were exposed to the environment for at least 12 h prior to initiating electrochemical testing. The open circuit potential (OCP) was performed for 30 min after which subsequent electrochemical tests in the sequence were performed. Electrochemical impedance spectroscopy (EIS) was performed from a frequency range of 10 kHz to 0.01 Hz with 10 mV AC amplitude and 10 points/decade. Mott–Schottky was performed from −1 V to the positive direction using a 40 mV step size and applying an excitation signal of 10 mV and frequency of 1000 Hz. The step size was selected to ensure limited variation in defect densities throughout the duration of the test^56^. Once the experiment concluded, the autoclave was cooled down to 25 °C and the system was depressurized and H_2_S was neutralized using the scrubber. The pH was measured after each test to ensure final measurements were 4 ± 0.2.

### Characterization after tests

To study the passive film composition after exposure, freshly polished samples were analyzed by X-ray photoelectron spectroscopy (XPS) after an immersion time of 24 h. The surface was characterized using an EnviroESCA XPS system utilizing a Mg X-ray source, pass energy of 40 eV, and step size 0.05 eV. A survey scan was done to identify elements present with high-resolution scans for Cr, Fe, Mo, Ni, C, S, and O also performed. Depth profile sputtering for 10, 20, and 50 min was performed using the IQE01 Ar^+^ gun equipped in the machine with the high current setting in the interface. The XPS fitting was performed using the XPSPEAK41software. To study the surfaces for evidence of localized corrosion, polished samples were immersed for one day and one week at the two tested temperatures after which they were cleaned thoroughly to perform SEM analysis.

### Supplementary information

Supplementary File: Additional information on X-ray photoelectron spectroscopy (XPS) fitting and energy dispersive spectroscopy (EDS) maps

## Data Availability

Data will be made available on request.
