# Supplementary material for: Localized corrosion in selective laser melted SS316L in CO2 and H2S brines at elevated temperatures
Source: Npj Mater Degrad. 2024 May 10;8(1):50. doi: 10.1038/s41529-024-00468-4 (PMC11087244; doi:10.1038/s41529-024-00468-4)
Supplement: Supplementary file 1 — Supplementary File: Additional information on X-ray photoelectron spectroscopy (XPS) fitting and energy dispersive spectroscopy (EDS) maps [file 41529_2024_468_MOESM1_ESM.pdf]

Supplementary File: Additional information on X-ray photoelectron spectroscopy (XPS) fitting and energy dispersive spectroscopy (EDS) maps

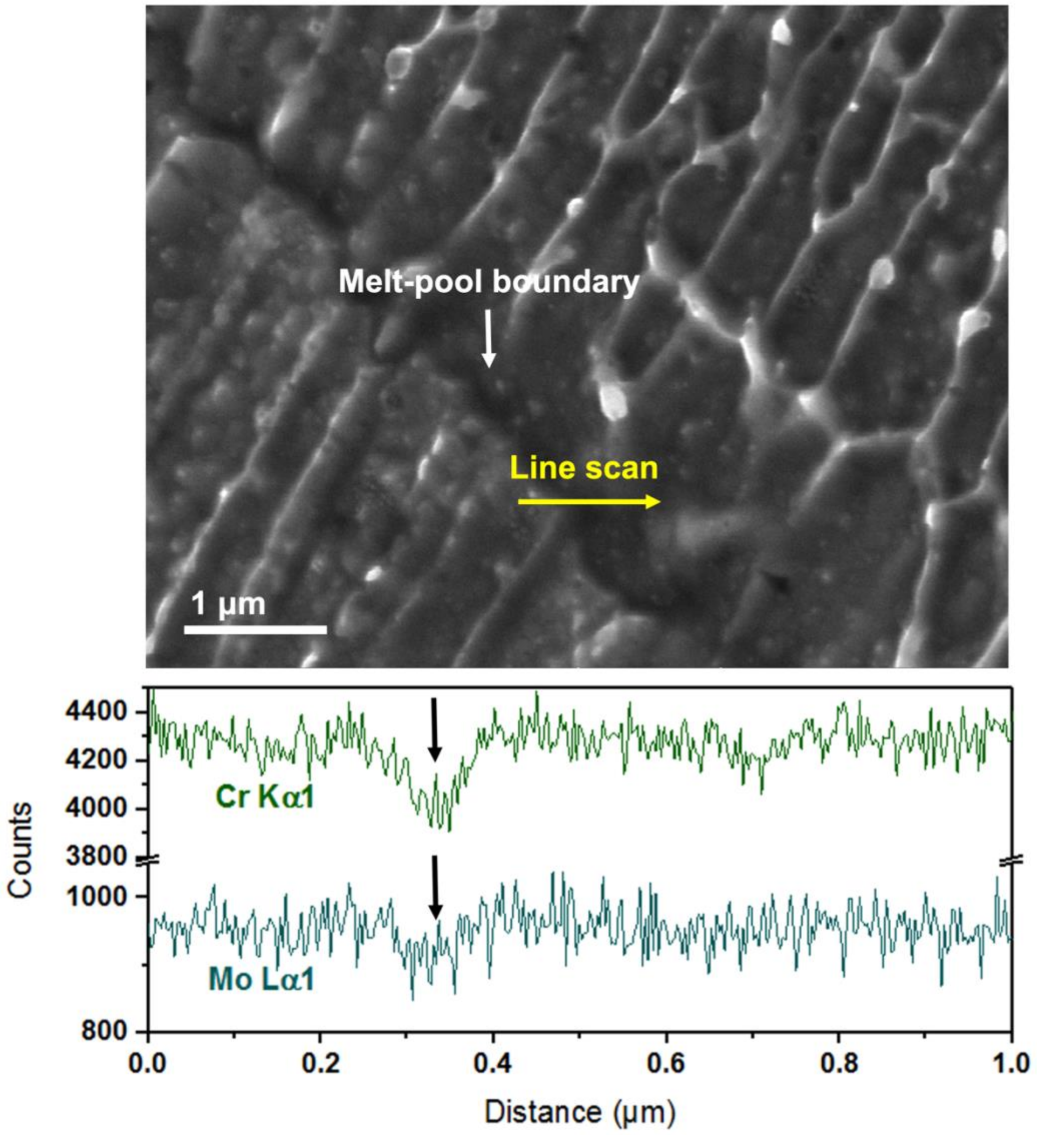

Supplementary Figure 1. SEM-EDS line scan showing the Cr and Mo depletion at the melt-pool boundary.

Supplementary Table 1. Composition of elements in the film measured from XPS spectra after 10, 20 and 50 minutes of sputtering.

Temperature (°C)	Sputter time (min)	Cr (at%)	Fe (at%)	Ni (at%)	Mo (at%)	S (at%)	O (at%)	C (at%)
25	10	9.79	10.27	2.87	2.68	5.27	39.6	29.52
	20	14.03	20.42	4.48	2.01	3.14	31.59	24.33
	50	11.8	26.13	5.29	1.83	2.45	24.51	27.99
125	10	7.54	5.39	4.36	4.98	23.47	17.4	36.85
	20	7.85	15.34	4.44	3.68	18.51	18.39	31.79
	50	7.09	18.96	3.69	3.31	12.07	14.78	40.1

Supplementary Table 2. XPS peak parameters of chemical species and the atomic percentage as a function of temperature for the 24h immersion samples.

**Element**	**Peak**	**Binding energy (eV)**	**25ºC (at%)**	**125ºC (at%)**
Cr $2p_{3/2}$	Cr metal	575.0	6.86	25.73
	Cr_2O_3	576.9	70.40	47.55
	$Cr(OH)_3$	577.4	22.74	26.72
Fe $2p_{3/2}$	Fe metal	707.3	34.18	38.46
	FeO	709.3	31.11	34.47
	Fe_2O_3	712.9	34.71	27.07
Mo 3d	S 2s	226.1	18.48	41.53
	Mo metal, $3d_{5/2}$	228.0	17.08	17.28
	MoS_2, $3d_{5/2}$	228.7	12.85	12.81
	Mo metal, $3d_{3/2}$	231.0	16.21	7.67
	MoS_2, $3d_{3/2}$	231.9	12.40	10.74
	MoO_3, $3d_{5/2}$	233.0	12.53	6.70
	MoO_3, $3d_{3/2}$	234.9	10.45	3.28
Ni 2p	Ni metal	853.1	73.11	53.95
	$Ni(OH)_2$	854.8	16.42	26.83
	$Ni(OH)_2$ satellite	859.1	10.47	19.21
O 1s	O^{2-}	530.6	46.08	24.15
	$(OH)^-$	531.7	53.92	75.85
S 2p	S_2^{2-}, $2p_{3/2}$	162.1	61.31	49.83
	S_2^{2-}, $2p_{1/2}$	163.6	38.69	50.17

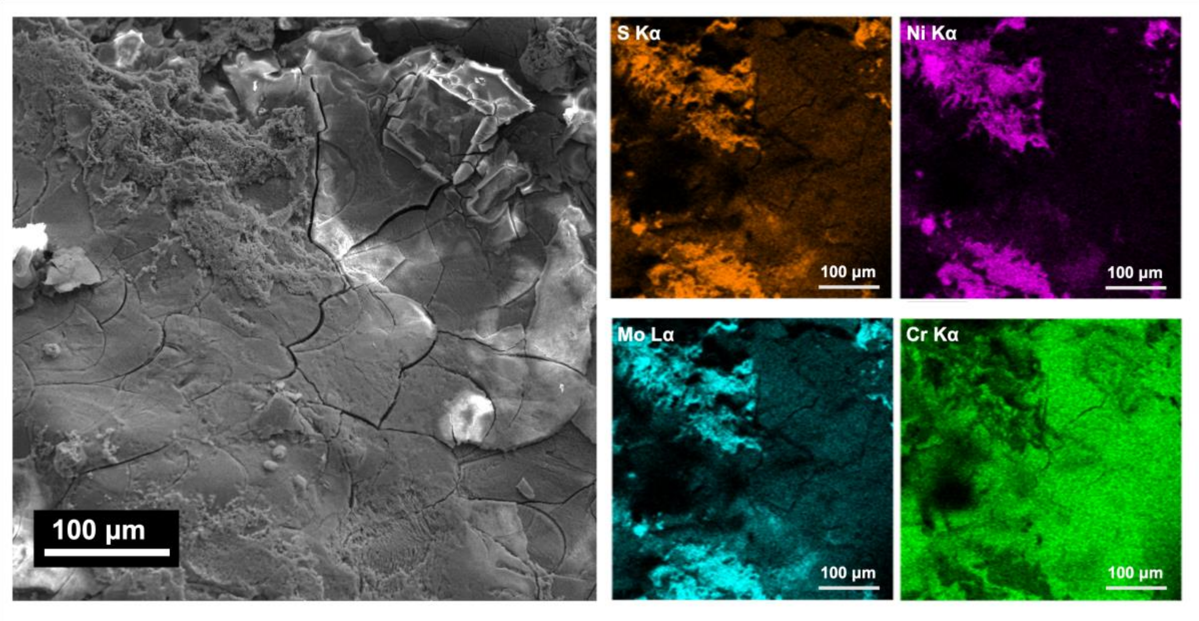

Supplementary Figure 2. EDS maps showing the Ni and Mo sulfides inside the pits after one week immersion at 125ºC.
